# Workplace exercise in telework: implementation of distance postural health actions in times of pandemic

**DOI:** 10.47626/1679-4435-2021-833

**Published:** 2021-12-30

**Authors:** Ana Carla de Freitas Caldas da Fonte, Vanessa Maria da Silva, Fabiola Lucchesi Rocha Carvalho, Graziella de Araujo Freitas

**Affiliations:** 1 Fisioterapia do Trabalho, Caixa de Assistência dos Funcionários do Banco do Brasil, Recife, PE, Brazil.; 2 Fonoaudiologia do Trabalho, Caixa de Assistência dos Funcionários do Banco do Brasil, Recife, PE, Brazil.; 3 Medicina do Trabalho, Caixa de Assistência dos Funcionários do Banco do Brasil, Brasília, DF, Brazil.; 4 Gerência de CliniCASSI, Caixa de Assistência dos Funcionários do Banco do Brasil, Recife, PE, Brazil.

**Keywords:** pandemics, occupational health, Internet-based intervention, physical therapy specialty, pandemias, saúde do trabalhador, intervenção baseada em Internet, fisioterapia

## Abstract

**Introduction:**

Telework, adopted by some companies as an alternative measure to ensure the continuity of services during the 2019 coronavirus disease pandemic, can have an impact on workers’ health, contributing to the onset of work-related musculoskeletal disorders and/or repetitive strain injuries. In this context, companies were required to develop strategies to ensure the implementation of occupational health actions through different means.

**Objectives:**

To discuss our experience of implementing a remote workplace exercise program.

**Methods:**

The experience report was based on the development and implementation of a remote workplace exercise intervention project for the employees of a bank who are working from home.

**Results:**

The remote workplace exercise program proved to be feasible, providing benefits in terms of saving resources, extending the reach of actions and increasing the number of employees involved, improving adherence of workers to health promotion programs, and enhancing their awareness of postural habits beyond the occupational setting.

**Conclusions:**

Interventions in occupational health and safety by technological means, associated with telework, can be seen as a new perspective during and after the pandemic, especially in banks, where remote work has proven to be efficient.

## Resumo

**Introdução:**

O regime de teletrabalho, adotado por algumas empresas como medida alternativa para continuidade dos serviços durante a pandemia causada pela doença do coronavírus 2019, pode gerar repercussões na saúde dos funcionários, contribuindo para o desencadeamento das doenças osteomusculares relacionadas ao trabalho e/ou lesões por esforços repetitivos. Nesse contexto, empresas precisaram criar estratégias para garantir a implantação de ações em saúde do trabalhador através de diferentes meios.

**Objetivos:**

Discutir a experiência de implementação de atividades de ginástica laboral remota.

**Métodos:**

O relato de experiência foi baseado na construção e implementação de um projeto de intervenção de ginástica laboral remota junto aos funcionários de uma instituição bancária que atuam em regime de teletrabalho.

**Resultados:**

A ginástica laboral remota se mostra viável, evidenciando economia de recursos, ampliação do alcance e quantidade de funcionários, melhor adesão dos trabalhadores a programas de promoção da saúde e conscientização sobre sua postura para além do âmbito ocupacional.

**Conclusões:**

As intervenções em saúde e segurança do trabalho por meios tecnológicos, associados ao teletrabalho, vêm se estabelecendo como uma nova perspectiva durante e após a pandemia, especialmente nas instituições bancárias, onde a jornada remota vem se revelando mais eficiente.

## INTRODUCTION

Occupational health encompasses a field of knowledge that aims to understand the relationship between work and the health/illness process, recognizing work as one of the determinants of this process. In Brazil, ordinance No. 1823, of August 2012, established the National Policy on Occupational Health, which, in its Article 2, ensures “comprehensive health care to workers, with an emphasis on surveillance, aimed at promoting and protecting workers’ health and at reducing morbidity and mortality resulting from development models and production processes.”^1^ Within this scenario, it is important to consider that working conditions and the performance of work activities can be a source of exposure to viruses and a territory for the spread of other (non-occupational) diseases, which have a direct or indirect impact on work activities.^2^

The current scenario of the 2019 coronavirus disease (COVID-19) pandemic, a disease caused by the novel severe acute respiratory syndrome coronavirus 2 (SARS-CoV-2), has mobilized several institutions and services due to its high transmissibility, symptom variability, lack of drug therapy, insufficient testing, severity of symptoms, and prolonged duration of clinical features and their possible consequences. Transmission occurs by close contact with infected individuals, by shaking hands, by touching contaminated objects or surfaces, such as cell phones, tables, doorknobs, toys, and computer keyboards, and by contact with saliva droplets through sneezing, coughing, and secretions, among others.^3^

With the recommendations of health agencies for social distancing during the COVID-19 pandemic, companies had to adapt to the new reality by placing their workers in a telework or home office working system.^4^ It is essential to understand the activities and conditions of telework in order to establish strategies to combat the pandemic and to continue the actions for protecting and promoting occupational health, while complying with the regulations and guidelines of the Brazilian Ministry of Health, Ministry of Labor, and other official regulatory bodies.

Telework was regulated by the 2017 labor reform in Brazil,^5^ and the equipment required for its execution needed to be defined based on a negotiation between employers and employees, including guidelines for the employees on how to avoid work-related accidents and illnesses. After declaring a state of public calamity and the need to adopt preventive actions in view of the high rates of contamination by the coronavirus, the government established alternative labor measures that included guidelines on telework.^6^ In light of this reality, many employees had to adapt the physical structure of their homes to work, since telework must be performed considering their daily working hours, with recess and lunch breaks. On the other hand, employers had to provide resources (physical and technological materials) to continue operating their services, in compliance with legislative guidelines.

The autonomy and variety of places where telework can be performed are organized by workers in their homes, leading to changes in the dynamics of time and space dimensions. Studies show different repercussions of telework on occupational health, including inadequate posture, musculoskeletal load, statistical load, cognitive requirements, work-related organizational and psychosocial factors, and diseases associated with the worker’s function. These conditions include work-related musculoskeletal disorders (WMSDs) and repetitive strain injuries (RSIs).^7^

Physical therapy, which studies the human movements during the performance of activities, has its actions based on its own therapeutic instruments, obtained through the study of biological, morphological, physiological, biochemical, biophysical, and biomechanical sciences, kinesiology, functional synergy, organ and system pathologies, and behavioral and social disciplines. Within this context, we can find occupational physical therapy, which, as an area of expertise, analyzes the adaptation of work to people through ergonomics and musculoskeletal disorders known as RSIs/WMSDs,^8^ focusing on the identification and correction of ergonomic risks, postural guidance, prevention, and health promotion through programs such as workplace exercise.^9,10^

Workplace exercise programs consist of specific exercises performed at the workplace, taking into account the activities and physical space available. In addition to improving musculoskeletal outcomes, workplace exercise aims to make workers aware of the need to change their lifestyle inside and outside the work environment through postural education.^11,12^

The benefits of implementing break exercise (or active breaks) during working hours have been reported and investigated by many authors. These breaks have been associated with greater willingness to work, motivation to adopt a healthier lifestyle, prevention of occupational diseases, and even increased productivity.^13-16^

According to Resolution No. 516 of March 23, 2020, physical therapists are authorized to provide care in the modalities of teleconsultation, teleconsulting, and telemonitoring in order to ensure opportunities for the continuity of treatment during the pandemic, without compromising health professionals’ and patients’ health.^17^

The current scenario calls for changes in the organization’s external environment, as well as the need to adapt it to a new context. In telework, it is assumed that workers may not follow health and safety rules either due to lack of information or difficulty in monitoring by employers and inspection bodies.^18^ Companies were required to develop strategies to ensure the implementation of occupational health programs and actions through different means, aiming to eliminate or minimize possible ergonomic risks during telework, as well as to raise awareness of work-related disorders, especially RSIs/WMSDs.

Therefore, this report aims to discuss our experience of implementing a remote workplace exercise program for the employees of a bank who are working from home during the COVID-19 pandemic.

## METHODS

Every intervention project must be thought of in an interdisciplinary manner, and the solutions and strategies must be developed by all involved actors. Aiming to ensure occupational health and safety in the current context, an interdisciplinary project was developed to implement a remote workplace exercise program aimed at the employees of a bank who are working from home.

The project was structured by health management specialists and physical therapists from a supplementary health insurance company that implements, together with the Specialized Service in Safety Engineering and Occupational Medicine (Serviço Especializado em Engenharia de Segurança e Medicina do Trabalho, SESMT), the Occupational Health Medical Control Program (Programa de Controle Médico de Saúde Ocupacional, PCMSO) in the bank. SESMT also has an interdisciplinary team consisting of an occupational physician, an occupational health nurse, a psychologist, and a speech therapist, all of whom also participate, directly or indirectly, in the planning and implementation of occupational health and safety actions at the institution.

The project was based on Regulatory Standards (Normas Regulamentadoras, NR) number 7,^19^ 9,^20^ and 17,^21^ of the Brazilian Ministry of Labor and Employment, and on Resolution No. 516 of March 23, 2020,^17^ of the Federal Board of Physical Therapy and Occupational Therapy. The project content was validated by the team and, after reaching a consensus, the activities were initiated.

To implement an intervention, it is important to consider its structuring components, which include resources, action processes, purposes, and environment.^22^

Box 1 summarizes the components required for the implementation of a remote workplace exercise program.

**Box 1:**
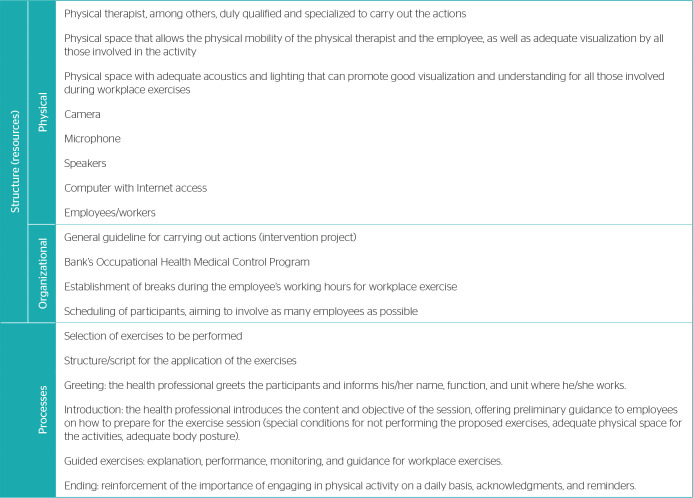
Components of the remote workplace exercise intervention

The target audience of the implemented remote workplace exercise program was employees from different departments of a bank, with branches located in different states of Brazil, who were working from home.

The exercise sessions were carried out as videoconference group meetings via Microsoft Teams® online platform. Invitation to join the event was sent via corporate electronic mailbox to employees, with pre-scheduled date and time on the platform. The program started in April 2020, with sessions twice a week, covering the morning and afternoon shifts; the program is still ongoing.

The workplace exercise sessions at this institution were conducted and guided in real time by specialized physical therapists, with the virtual meetings being held twice a week, lasting 15 minutes each. Approximately 20 employees participated in each session.

Physical therapists rotated according to a predetermined schedule, on different days, shifts, and times, aiming at involving most employees in the different work shifts.

The physical therapist in charge pre-selected the exercises by considering an integral approach to the musculoskeletal system through compensatory exercises. The choice and order of execution of exercises were based on technical and scientific aspects.

Compensatory exercises are those performed during working hours, in the middle of the work shift, or at peak fatigue time and take into account the activities and physical demands of the work performed, such as repetitive movements, inadequate postures, and most prevalent complaints. These exercises aim to prevent workers’ habitual poor posture and to achieve an active antagonist muscle balance by exercising the muscles less used during the workday and relaxing the muscles most used during routine tasks, thus reducing fatigue.^13,23^

Based on the foregoing concepts, the proposed exercises involved the following aspects: a) active kinesiotherapy; b) static and dynamic stretching; c) muscle strengthening; and d) muscle relaxation.

The number of exercises varied according to the body regions to be exercised, the functional goals, and the possibility of working different aspects in the same exercise. Each session began with a set of warm-up exercises at 10 repetitions each, always involving the lower limbs, upper limbs, and spine. Stretching exercises focused on the upper limbs and spine, especially the lumbar and cervical regions, at one repetition of 20-30 seconds each. Strengthening exercises always targeted the upper limbs and spinal extensor muscles and consisted of one or two sets at 10 to 15 repetitions each. Muscle relaxation exercises were performed by combining upper limb exercises with breathing exercises, ranging from 5 to 10 repetitions each.

## Results and discussion

We have long witnessed the impact that a pandemic can exert at the biomedical, epidemiological, social, cultural, economic, and political level in the world. This becomes evident when we analyze health in the occupational setting, as an environment that brings together all these aspects in a multidimensional manner, under a broader perspective. It is important to understand and learn how to deal with the physical and psychological damage^24^ that will persist during and after the pandemic.

It is important to consider that, with the advent of new technological work tools, combined with new organizational forms, work relationships, and care pathways that have emerged during the pandemic, new methodologies for actions in occupational health are being developed and implemented. Some changes that have been made tend to be consolidated in certain sectors, making the urgency of innovation evidenced by the pandemic.^25^

Some companies, along with their employees, have already been using technological platforms as tools in the implementation of occupational health and safety actions, including workplace exercise programs delivered online by specialized health professionals and training in ergonomics, available through virtual learning environments,^26^ health education portals, and applications.

One of the positive aspects of remote workplace exercise is its feasibility, that is, the likelihood of an intervention being successful, considering its constituent components, context, and the methodologies used. This aspect has been demonstrated in a previous study,^27^ with evidence that every technology-mediated intervention must consider its applicability, feasibility, optimization of resources, and minimization of errors.

This type of intervention also led to an increase in the voluntary adherence of employees to the exercise program, thus contributing to extending the reach of actions and increasing the number of participants. This may also be explained by the decentralization of the activities, as the exercise sessions were made available to different states and to all employees in telework, regardless of their function.

Also, workplace exercise contributes to adopting healthy postural habits and experiencing improvements in quality of life, both inside and outside the occupational setting.^28^ This fact was evidenced by the positive feedbacks, given to managers by employees, regarding their perceptions of body awareness and muscle flexibility before and after workplace exercise sessions.

It is important to highlight that, for an effective prevention of RSIs/WMSDs, remote workplace exercise programs must be implemented together with other actions, such as health education through lectures, training sessions, and campaigns, among other ergonomic measures. Furthermore, there should be continuous investment in personnel training, equipment maintenance, and motivation and incentive policies, regardless of the working system. Altogether, these measures contribute to the improvement of the organizational climate, effectiveness of interventions, and reduction of unnecessary costs.^29^

Recently, the real-time exercise sessions were recorded and made available to employees on a weekly basis, aiming to make the exercise part of their daily routine, as well as to increase workers’ participation throughout the country. In addition, methods to monitor and evaluate the effectiveness of this type of intervention are being discussed by the team, also considering the employees’ opinions.

## CONCLUSIONS

Interventions in occupational health and safety by technological means, associated with telework, can be seen as a new perspective during the pandemic, especially in banks, where remote work has proven to be productive and efficient.

In the current setting, a remote workplace exercise program was able to ensure occupational health and safety for employees in telework, while complying with sanitary and legal requirements of the pandemic, in light of the provisions of the PCMSO.

This intervention method was positively evaluated by all those involved. It also provided benefits in terms of feasibility, saving resources, extending the reach of actions and increasing the number of employees involved, improving adherence of workers to health promotion programs, and enhancing their awareness of postural habits beyond the occupational setting.
